# Supplementary material for: Nanometric Cu-ZnO Particles Supported on N-Doped Graphitic Carbon as Catalysts for the Selective CO2 Hydrogenation to Methanol
Source: Nanomaterials (Basel). 2024 Mar 6;14(5):476. doi: 10.3390/nano14050476 (PMC10934795; doi:10.3390/nano14050476)
Supplement: Supplementary file 1 [file nanomaterials-14-00476-s001.zip › nanomaterials-2855322-supplementary.pdf]

Supporting Information

Nanometric Cu-ZnO Particles Supported on N-Doped Graphitic Carbon as Catalysts for the Selective CO_2 Hydrogenation to Methanol

Table S1. List of metal amounts employed in the preparation of Cu@(N)C (samples **1**) and Cu-ZnO@C (samples **2**-**6**) under study.

Samples No.	$C_{Cu(OAc)2}$ (mol/L)	$m_{Zn(OAc)2}$ (mg)
**1**	0.005	-
**2**	0.005	109.8
**3**	0.005	43.9
**4**	0.005	22.0
**5**	0.005	27.3
**6**	0.005	11.0

Table S2. Analytical data of sample **4** based on XPS analysis of the surface.

Element	% Mass	% Atomic
Cu2p	0.999	0.199
O1s	9.405	7.440
C1s	84.783	89.342
N1s	2.940	2.657
Zn2p	1.873	0.363

Estimation of the diffusion coefficients.

The reaction mixture has been treated as a hexa-component (CO_2, H_2, CH_4, CO, CH_3OH, H_2O) real gas. The calculations were performed using an algorithm described in a previous report from of our group [5]. The calculated values of the diffusion coefficients for the reactants in the gas reaction mixture ($D_{CO2,m}$ and $D_{H2,m}$) are presented in Table S3.

Calculation of the Weisz-Prater number:

The adimensional Weisz-Prater criterion [6, 7] is given by:

$$N_{W-P} = \frac{r\rho_{cat} R_p^2}{C_s D_{eff}} \leq 0.3 \qquad \textbf{(S1)}$$

where:

r - reaction rate expressed per unit mass of catalyst, $mol \cdot s^{-1} \cdot (g^{-1})_{cat}$

ρ_{cat} - packed catalyst density, $g \cdot cm^{-3}$

R_p - catalyst particle radius, cm

C_s - concentration of the reactant at the surface of the catalyst particles, mol·cm^{-3}

D_{eff} - effective diffusion coefficient, cm^2·s^{-1}

The average catalyst particle size considered for the calculations was 200 μm. Packed catalyst densities were estimated by weighting a known volume (around 2 mL) of solid catalyst, the obtained values are (in order, from catalyst sample **1** to **6**): 0.119; 0.110; 0.106, 0.112, 0.121 and 0.120 g/cm^3.

The concentration of the reactants on the catalyst surface is considered the same as in the gas mixture due to the space velocity values used during the experiments. Critical temperature, pressure and volume (Tc, Pc, Vc) values were used to determine the *a* and *b* van der Waals constants of pure gases that were summed according to the mixing rules described by Hirschfelder at al.[8] to obtain the *a* and *b* constants of the hexa-component gas reaction mixtures. The molar volume was further calculated from the van der Waals equation of state for the experimental conditions (P, T) of each catalytic test, taking into account the gas-phase composition inside the reactor (determined by gas-chromatography). Slightly larger values compared to the application of the ideal gas equation of state were obtained, thus indicating a very small prevalence of the repulsive intermolecular forces. The ratio between the mole fraction of the reactant and the molar volume results in the reactant concentrations in listed in Table S3.

All values of the Weisz-Prater criterion (Table S3) are by far smaller than 0.3

indicating that no mass transfer limitations affect the ongoing catalytic reactions.

Table S3: Values of the gas-phase reactant concentration (C), diffusion coefficients (D) of the reactants in the hexa-component gas mixture, average CO_2 transformation rate ($\overline{r}$) and Weisz-Prater criterion (N) for P = 40 bar, CO_2:H_2 (vol) = 1:4, total flow rate 4 mL/min as a function of the reaction temperature.

sample	T (^{O}C)	$C_{CO2}\cdot10^4$ (mol/cm^3)	$C_{H2}\cdot10^4$ (mol/cm^3)	$D_{CO2,m}$ (cm^2/s)	$D_{H2,m}$ (cm^2/s)	$\overline{-r_{CO_2}}$ (mol CO_2/ (s·g$_{(Cu+Zn) cat}$))	$\overline{-r_{CO_2}}$ (mol CO_2/ (s·mol$_{(Cu+Zn) cat}$))	N_{W-P} CO_2	N_{W-P} H_2
**1**	150	2.27	9.09	0.035	0.142	0	0	--	--
	200	2.03	8.12	0.043	0.172	$1.93\cdot10^{-8}$	$1.56\cdot10^{-5}$	$5.17\cdot10^{-18}$	$1.29\cdot10^{-18}$
	250	1.66	6.68	0.050	0.203	$1.41\cdot10^{-7}$	$1.14\cdot10^{-4}$	$3.93\cdot10^{-17}$	$9.70\cdot10^{-18}$
	300	2.19	9.01	0.056	0.233	$6.01\cdot10^{-7}$	$4.84\cdot10^{-4}$	$1.13\cdot10^{-16}$	$2.66\cdot10^{-17}$
**2**	150	2.02	8.11	0.035	0.141	$9.38\cdot10^{-8}$	$4.27\cdot10^{-5}$	$2.83\cdot10^{-17}$	$7.05\cdot10^{-18}$
	200	1.66	6.69	0.042	0.171	$1.82\cdot10^{-7}$	$8.28\cdot10^{-5}$	$5.54\cdot10^{-17}$	$1.36\cdot10^{-17}$
	250	2.12	8.86	0.045	0.189	$1.25\cdot10^{-6}$	$5.69\cdot10^{-4}$	$2.81\cdot10^{-16}$	$6.38\cdot10^{-17}$
	300	1.61	7.62	0.044	0.205	$3.32\cdot10^{-6}$	$1.51\cdot10^{-3}$	$1.00\cdot10^{-15}$	$1.82\cdot10^{-16}$

**3**	150	1.84	7.34	0.035	0.142	0	0	--	--
	200	1.66	6.68	0.042	0.171	$1.62\cdot10^{-6}$	$1.04\cdot10^{-4}$	$3.48\cdot10^{-17}$	$8.53\cdot10^{-18}$
	250	1.57	6.53	0.045	0.189	$1.21\cdot10^{-5}$	$7.73\cdot10^{-4}$	$2.58\cdot10^{-16}$	$5.88\cdot10^{-17}$
	300	1.83	8.55	0.045	0.206	$3.11\cdot10^{-5}$	$1.99\cdot10^{-3}$	$5.70\cdot10^{-16}$	$1.06\cdot10^{-16}$
**4**	150	2.02	8.10	0.035	0.140	$1.51\cdot10^{-7}$	$9.30\cdot10^{-5}$	$2.44\cdot10^{-17}$	$6.07\cdot10^{-18}$
	200	1.82	7.30	0.042	0.169	$2.94\cdot10^{-7}$	$1.81\cdot10^{-4}$	$4.42\cdot10^{-17}$	$1.08\cdot10^{-17}$
	250	1.74	7.23	0.047	0.196	$1.00\cdot10^{-6}$	$6.14\cdot10^{-4}$	$1.40\cdot10^{-16}$	$3.22\cdot10^{-17}$
	300	1.32	6.27	0.044	0.204	$3.92\cdot10^{-6}$	$2.41\cdot10^{-3}$	$7.78\cdot10^{-16}$	$1.39\cdot10^{-16}$
**5**	150	2.27	9.09	0.035	0.141	$3.04\cdot10^{-7}$	$1.94\cdot10^{-5}$	$7.64\cdot10^{-18}$	$1.90\cdot10^{-18}$
	200	2.02	8.11	0.042	0.171	$1.06\cdot10^{-6}$	$6.79\cdot10^{-5}$	$2.50\cdot10^{-17}$	$6.19\cdot10^{-18}$
	250	1.78	7.24	0.048	0.196	$6.38\cdot10^{-6}$	$4.07\cdot10^{-4}$	$1.55\cdot10^{-16}$	$3.61\cdot10^{-17}$
	300	1.50	6.41	0.049	0.212	$1.88\cdot10^{-5}$	$1.20\cdot10^{-3}$	$5.11\cdot10^{-16}$	$1.12\cdot10^{-16}$
**6**	150	1.67	6.69	0.035	0.141	$4.76\cdot10^{-8}$	$3.10\cdot10^{-5}$	$9.71\cdot10^{-18}$	$2.42\cdot10^{-18}$
	200	2.27	9.08	0.043	0.171	$4.76\cdot10^{-8}$	$3.10\cdot10^{-5}$	$5.90\cdot10^{-18}$	$1.47\cdot10^{-18}$
	250	1.98	8.05	0.049	0.199	$4.54\cdot10^{-7}$	$2.95\cdot10^{-4}$	$5.67\cdot10^{-17}$	$1.36\cdot10^{-17}$
	300	1.82	7.86	0.050	0.219	$1.69\cdot10^{-6}$	$1.10\cdot10^{-3}$	$2.21\cdot10^{-16}$	$4.73\cdot10^{-17}$

The average CO_2 transformation rates are calculated taking into account only the metal load of the catalysts (neglecting the carbonaceous support). Table S3 lists two series of values: one expressed in moles of CO_2 transformed per unit time and per grams of metal (used in the calculation of the Weisz-Prater criterion) and another one where the amount of metal is expressed in moles (becoming thus an average rate expressed in s^{-1}, which corresponds to the average turnover frequency - TOF).

Calculation of the mixture composition at the equilibrium.

Equilibrium data have been determined through the minimization of the Gibbs free energy of the system using a RGIBBS module in AspenPlus® considering the Soave-Redlich-Kwong Equation of State without additional corrections on fugacity coefficients. Only methanol synthesis and reverse water gas shift reaction were considered as CO_2 conversion paths. The results are presented in Table 2 of the main text.

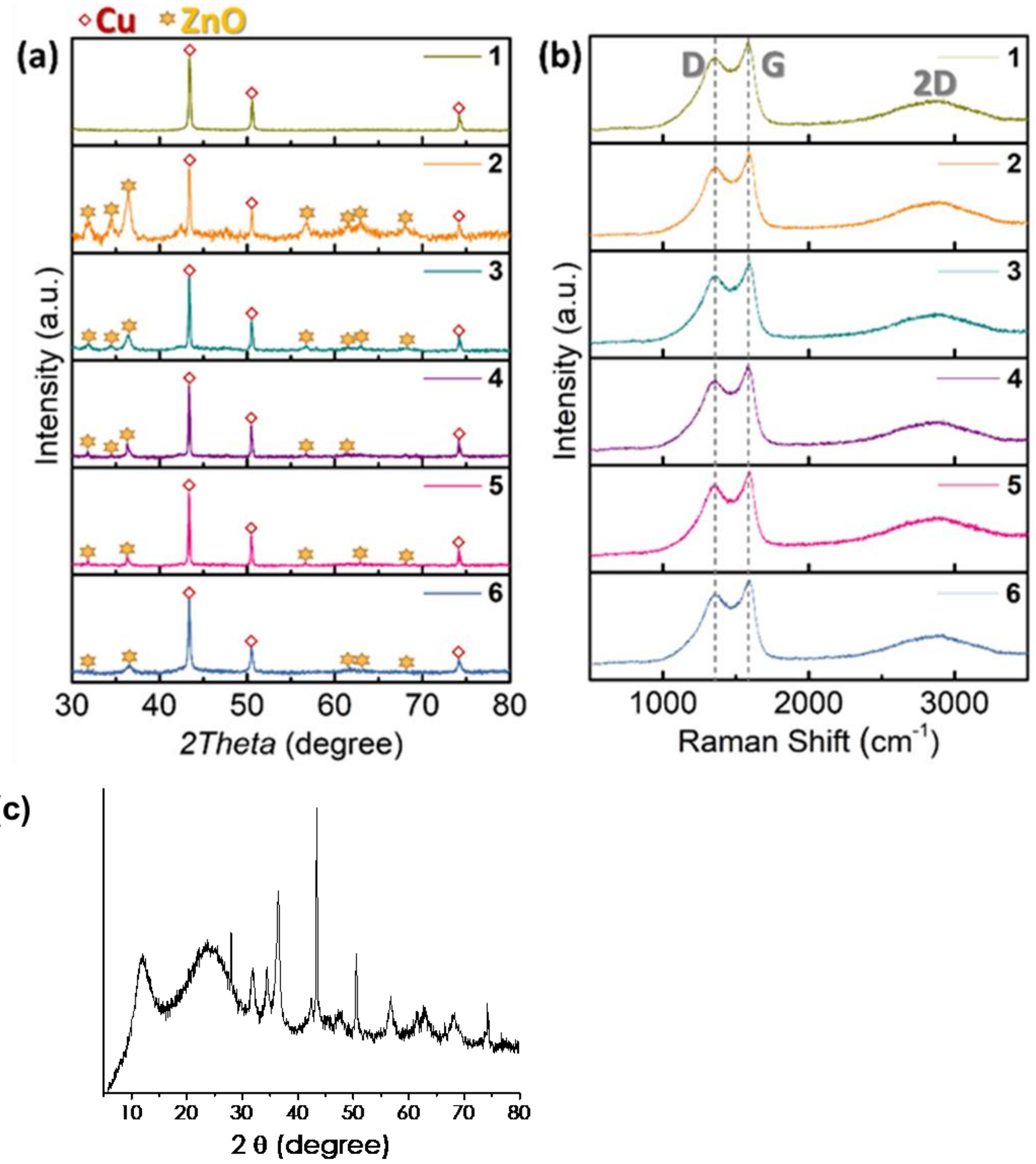

Figure S1. XRD patterns (**a**) and Raman spectra (**b**) of the samples **1**-**6**. Symbols: Cu (◇) and ZnO (✶). Full XRD pattern of Cu-ZnO@(N)C (**c**).

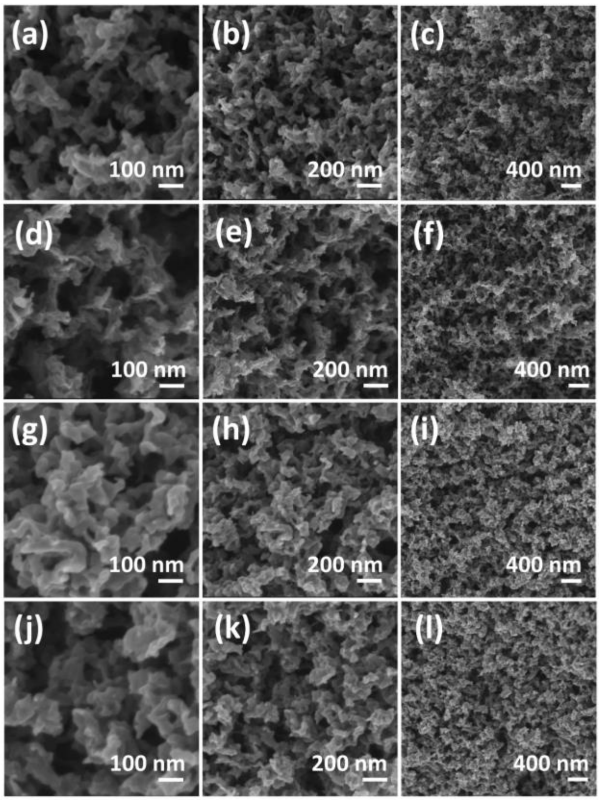

Figure S2. HR-FESEM images of samples **3 (a-c), 4** (**d**-**f**), **5** (**g**-**i**) and **6 (j**-**l**)

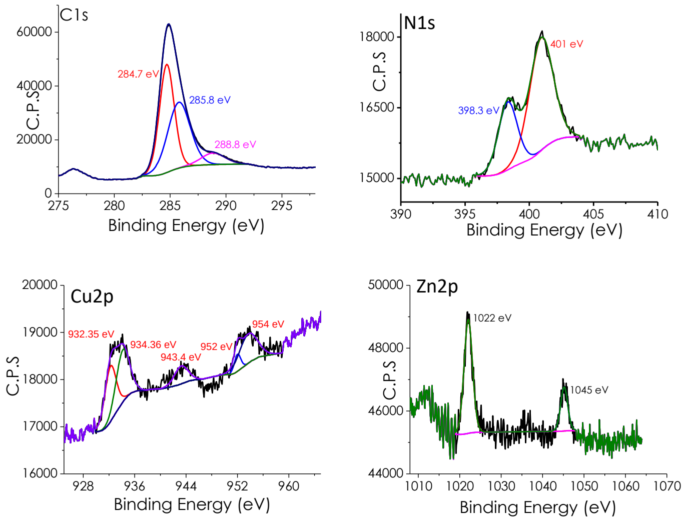

Figure S3. High resolution XPS peaks and their best deconvolution to individuals components measured for sample **4**.

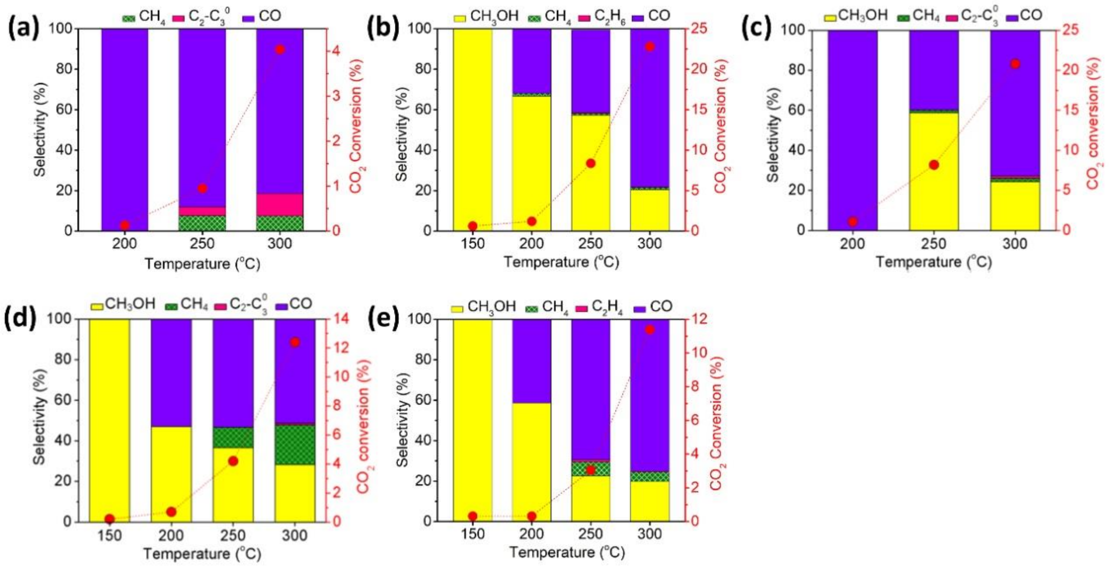

Figure S4. CO_2 conversion and selectivity for samples **1** (**a**), **2** (**b**), **3** (**c**), **5** (**d**) and **6** (**e**) at different temperatures. Reaction conditions: H_2/CO_2 ratio of 4, total flow 4 mL/min, 40 bar, 40 mg catalyst.

Table S4. CO_2 conversion and selectivity by the 316 SS austenite stainless steel reactor in the absence of catalyst at different temperature.

T (°C)	**C (%)**	S (%)	S (%)	**S (%)**
	**CO_2**	CH_4	CO	**CH_3OH**
150	**0**	-	-	**-**
200	**0.1**	25.9	74.1	**0**
250	**0.4**	10.9	89.1	**0**
300	**1.1**	7.6	92.4	**0**

Table S5. Catalytic activity of Cu-ZnO/Al_2O_3 in the range of temperatures from 150 to 300 °C and methanol selectivity at 300 °C over the time. Reaction conditions: H_2/CO_2 ratio of 4, total flow 4 mL/min, 40 bar, 40 mg catalyst.

T (°C)	**C (%) CO_2**	S (%) CH_4	S (%) C_2H_6	S (%) C_3H_8	S (%) *n*-C_4H_{10}	S (%) C_2H_4	S (%) C_3H_6	S (%) CH_3OH	S (%) CO
150	**0.17**	0	0	0	0	0	0	100	0
200	**0.34**	0	0	0	0	0	0	72.46	27.54
250	**3.58**	3.12	0.98	0	0	0	0	58.66	37.24
300	**12.28**	2.05	0.63	0	0	0	0	33.09	64.23

Catalytic activity of CuZn/Al_2O_3-imp

	**CuZn/Al2O3-imp**								
T (°C)	**C (%) CO2**	S (%) CH4	S (%) C2H6	S (%) C3H8	S (%) *n*-C4H10	S (%) C2H4	S (%) C3H6	S (%) CH3OH	S (%) CO
150	**0**	--	--	--	--	--	--	--	--
200	**0.09**	0	0	0	0	0	0	0	100
250	**0.74**	15.98	0	0	0	0	0	0	84.02
300	**3.20**	27.62	0	0	0	0	0	0	72.38

Reaction conditions: 40 mg 4:1 H_2:CO_2 (3.2 mL/min. H_2, 0.8 mL/min CO_2) 40 bar

Stability test showing methanol selectivity decrease over time up to a value of 25 %,

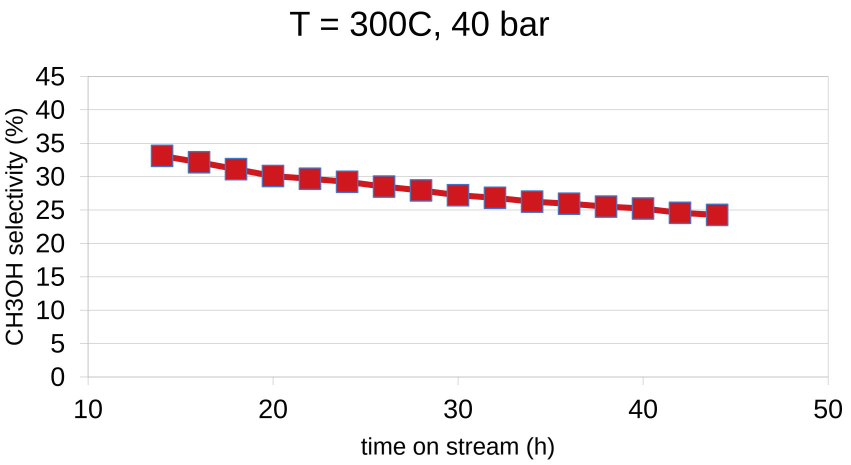

Figure S5. Photographs of fresh and spent Cu-ZnO/Al_2O_3 catalyst. The carbon content of the spent Cu-ZnO/Al_2O_3 was over 1 %.

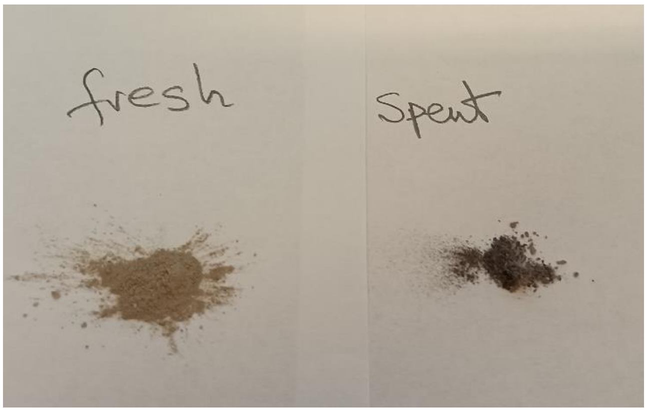

Estimation of the apparent activation energy:

From the transformation rates listed in Table S2, Arrhenius plots can be traced allowing to evaluate the values of the apparent activation energy for each catalyst studied (**Figure S4**).

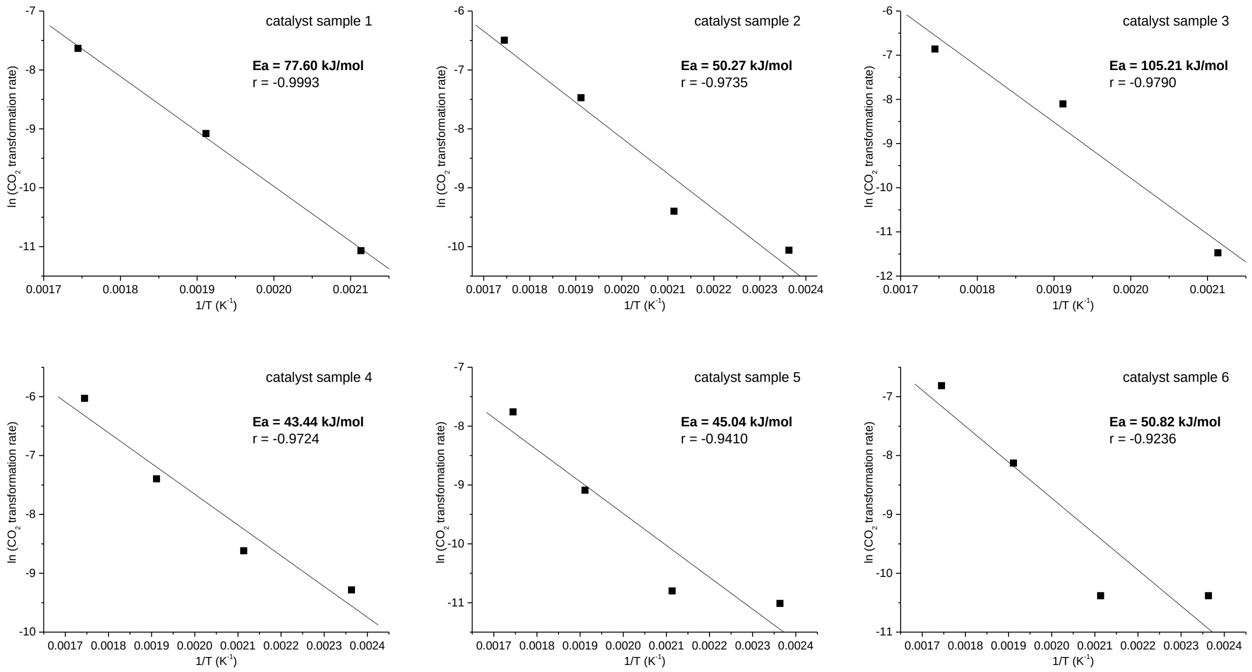

Figure S6. Arrhenius plots for catalysts **1** to **6**; the value of the apparent activation energy is indicated on each graph, together with the correlation coefficient of the linear regression.

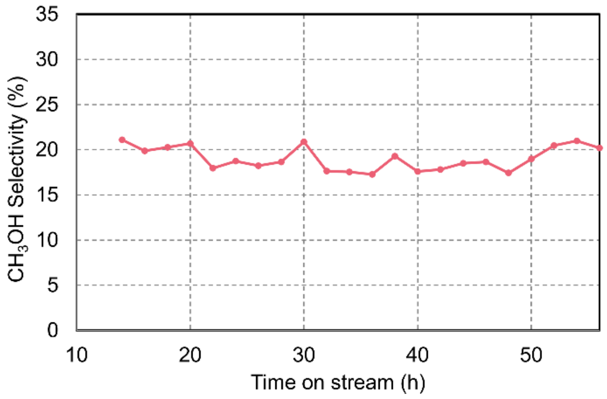

Figure S7. Stability test of sample **4** at 300 °C. Reaction conditions: H_2/CO_2 ratio of 4, total flow 4 mL/min, 40 bar, 40 mg catalyst.

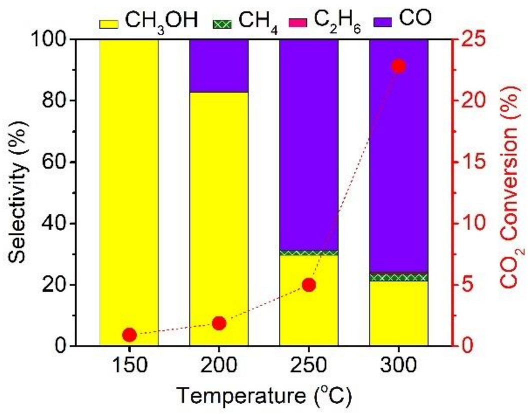

Figure S8. CO_2 conversion and selectivity for sample **4** at different temperatures after a long-time stability test. Reaction conditions: H_2/CO_2 ratio of 4, total flow 4 mL/min, 40 bar, 40 mg catalyst.

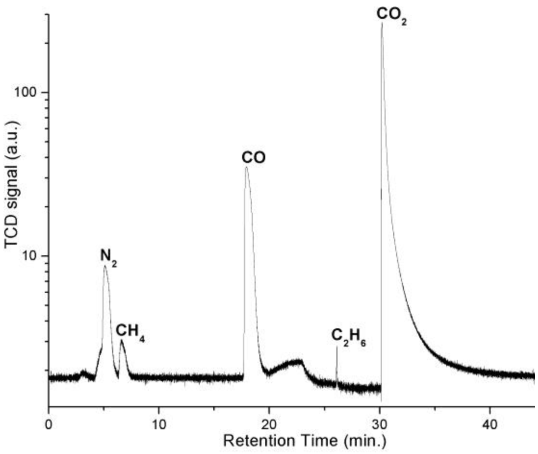

Chart S1 a) CG analysis (Restek MolSieve 5A PLOT column, TCD detection, H_2 carrier gas) for catalyst sample 4 after 68 h Time on Stream (experiment at 300ºC, 40 bar, H_2:CO_2 = 3.2:0.8 mL/min)

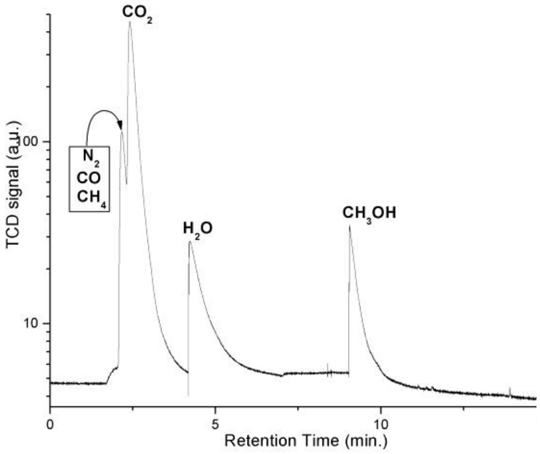

Chart S1 b) CG analysis (Supelco SupelQ PLOT column, TCD detection, H_2 carrier gas) for catalyst sample 4 after 68 h Time on Stream (experiment at 300ºC, 40 bar, H_2:CO_2 = 3.2:0.8 mL/min)

Table S6. CO_2 conversion and selectivity for sample **1** at different temperature.

T (°C)	**C (%) CO_2**	**S (%) CH_4**	S (%) C_2H_6	S (%) C_3H_8	S (%) CO	**S (%) CH_3OH**
150	**-**	-	-	-	-	**-**
200	**0.13**	0	0	0	100	**0**
250	**0.95**	7.64	1.32	3.0	88.04	**0**
300	**4.04**	7.48	5.03	6.06	81.43	**0**

Table S7. CO_2 conversion and selectivity for sample **2** at different temperature.

T (°C)	**C (%) CO_2**	**S (%) CH_4**	S (%) C_2H_6	S (%) C_3H_8	S (%) CO	**S (%) CH_3OH**
150	**0.63**	0	0	-	-	**100**
200	**1.22**	1.36	0	0	5.43	**66.71**
250	**8.38**	0.80	0.52	0	40.85	**57.31**
300	**22.28**	1.07	0.31	0	78.18	**20.44**

Table S8. CO_2 conversion and selectivity for sample **3** at different temperature.

T (°C)	**C (%) CO_2**	**S (%) CH_4**	S (%) C_2H_6	S (%) C_3H_8	S (%) CO	**S (%) CH_3OH**
150	**0.2**	0	0	0	0	**100**
200	**0.7**	0	0	0	52.9	**47.1**
250	**4.2**	10.1	0	0	53.3	**36.6**
300	**12.4**	19.6	1.0	0	51.3	**28.2**

Table S9. CO_2 conversion and selectivity for sample **4** at different temperature.

T (°C)	**C (%)**	**S (%)**	S (%)	S (%)	S (%)	**S (%)**
	**CO_2**	**CH_4**	C_2H_6	C_3H_8	CO	**CH_3OH**
150	**0.89**	0	0	0	0	**100**
200	**1.73**	0	0	0	10.26	**89.74**
250	**5.88**	0	1.52	0	73.57	**24.91**
300	**23.06**	1.01	0.49	0	77.49	**21.00**

Table S10. CO_2 conversion and selectivity for sample **5** at different temperature.

T (°C)	**C (%)**	**S (%)**	S (%)	S (%)	S (%)	**S (%)**
	**CO_2**	**CH_4**	C_2H_6	C_3H_8	CO	**CH_3OH**
150	**0.2**	0	0	0	0	**100**
200	**0.2**	0	0	0	52.9	**47.1**
250	**1.2**	10.1	0	0	53.3	**36.6**
300	**4.4**	19.6	1.0	0	51.3	**28.2**

Table S11. CO_2 conversion and selectivity for sample **6** at different temperature.

T (°C)	**C (%)**	**S (%)**	S (%)	S (%)	S (%)	**S (%)**
	**CO_2**	**CH_4**	C_2H_6	C_3H_8	CO	**CH_3OH**
150	**0.32**	0	0	0	0	**100**
200	**0.32**	0	0	0	41.37	**58.63**
250	**3.05**	6.91	1.06	0	69.44	**22.60**
300	**11.39**	4.68	0.36	0	75.13	**19.82**

Table S12. CO_2 conversion and selectivity for sample **4** at different temperature after

a long-time stability test.

T (^oC)	**C (%) CO_2**	**S (%) CH_4**	S (%) C_2H_6	S (%) C_3H_8	S (%) CO	**S (%) CH_3OH**
150	**0.92**	0	0	0	0	100
200	**1.87**	0	0	0	17.14	82.86
250	**4.99**	1.45	0	0	68.91	29.64
300	**22.83**	2.30	0.52	0	75.91	21.28

Table S13. Comparison of the performance of catalyst **4** with that of other catalysts reported in the literature for methanol synthesis from CO_2.

Catalyst	Reaction conditions	Methanol productivity $g_{CH3OH} \times kg_{catalyst}^{-1} \times h^{-1}$	Ref.
Cu-ZnO/(N)C	40 bar, 300 °C, CO_2/H_2 1/4, 6000 $mL \times g^{-1} \times h^{-1}$. Stable for longer than 54 h	83	This work
Cu-ZnO/Al_2O_3	40 bar, 300 °C, CO_2/H_2 1/4, 6000 $mL \times g^{-1} \times h^{-1}$	67[a]	This work
Cu/CeO_2	30 bar, 250 °C, CO_2/H_2 1/3, 30000 $mL \times g^{-1} \times h^{-1}$	45	[9]
ZnO_x/ZrO_2	20 bar, 300 °C, CO_2/H_2 1/3, 9000 $mL \times g^{-1} \times h^{-1}$	120	[10]
Cu/ZrO_2	30 bar, 230 °C, CO_2/H_2 1/3, 50000 $mL \times g^{-1} \times h^{-1}$	17[b]	[11]
In_2O_3	40 bar, 200 °C, CO_2/H_2 1/4, 2000 $mL \times g^{-1} \times h^{-1}$	4	[12]
CuZnZr/$CuBr_2$	50 bar, 250 °C, CO_2/H_2 1/3, 3000 $mL \times g^{-1} \times h^{-1}$	100[b]	[13]

CuZnAlCe	30 bar, 250 °C, CO_2/H_2 1/3, 12000 mL×g^{-1}×h^{-1}	213	[14]

[a] Activity of the fresh catalyst. Deactivates over the time. [b]Estimated from reported data.

Table S14. Hirshfeld charge distribution of Cu atoms in Cu_{13} and Cu_{13} adsorbed on pyridinic-N atom of N-doped graphene (Cu_{13}/N-C). Cu atoms numbering as in Figure S1.

No.	1	2	3	4	5	6	7	8	9	10	11	12	13	sum
Cu_{13}	0	0	0	0	0	0	0	0	0	0	0	0	0	0
Cu_{13}/N-C	0	0	-0.01	0	-0.01	0.01	0.13	0.12	0.09	0.1	0.13	-0.01	0.2	0.75

Table S15. Energy barriers of transition states (TS) in the RWGS+CO+hydro and formate pathways. TS labels according to Figure 2.

	**RWGS+CO+hydro**	**Ea (eV)**	**Formate**	**Ea (eV)**
TS1	$*CO_2+*H+5/2H_2 \rightarrow$ $*HOCO+5/2H_2$	1.89	$*CO_2+*H+5/2H_2 \rightarrow$ $*HCOO+5/2H_2$	0.35
TS2	$*HOCO+5/2H_2 \rightarrow$ $*HO+*CO+5/2H_2$	2.01	$*HCOO+*H+2H_2 \rightarrow$ $*HCOOH+2H_2$	2.12
TS3	$*HO+*CO+*H+2H_2 \rightarrow$ $*H_2O+*CO+2H_2$	1.87	$*HCOOH+*H+3/2H_2 \rightarrow$ $*H_2COOH+3/2H_2$	0.79
TS4	$*CO+*H+H_2O+3/2H_2 \rightarrow$ $*HCO+H_2O+3/2H_2$	0.78	$*H_2COOH+3/2H_2 \rightarrow$ $*H_2CO+*OH+3/2H_2$	1.10
TS5	$*HCO+*H+H_2O+H_2 \rightarrow$ $*H_2CO+H_2O+H_2$	1.91	$*H_2CO+*OH+*H+H_2 \rightarrow$ $*H_2CO+*H_2O+H2$	1.77
TS6	$*H_2CO+*H+H_2O+1/2H_2 \rightarrow$ $*H_3CO+H_2O+1/2H_2$	3.11	$*H_2CO+*H+H_2O+1/2H_2 \rightarrow$ $*H_3CO+H_2O+1/2H_2$	0.84
TS7	$*H_3CO+*H+H_2O \rightarrow$ $*CH_3OH+H_2O$	1.92	$*H_3CO+*H+H_2O \rightarrow$ $*CH_3OH+H_2O$	1.56

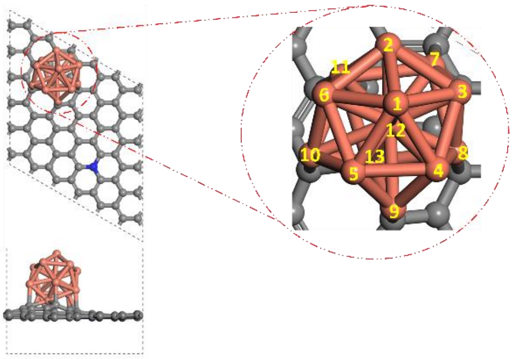

Figure S9. Atom numbering in the Cu_{13} cluster. The model shows the adsorption of the Cu_{13} cluster on pyridinic-N atom of N-doped graphene. Brown, black and blue represent Cu, C and N atoms, respectively.

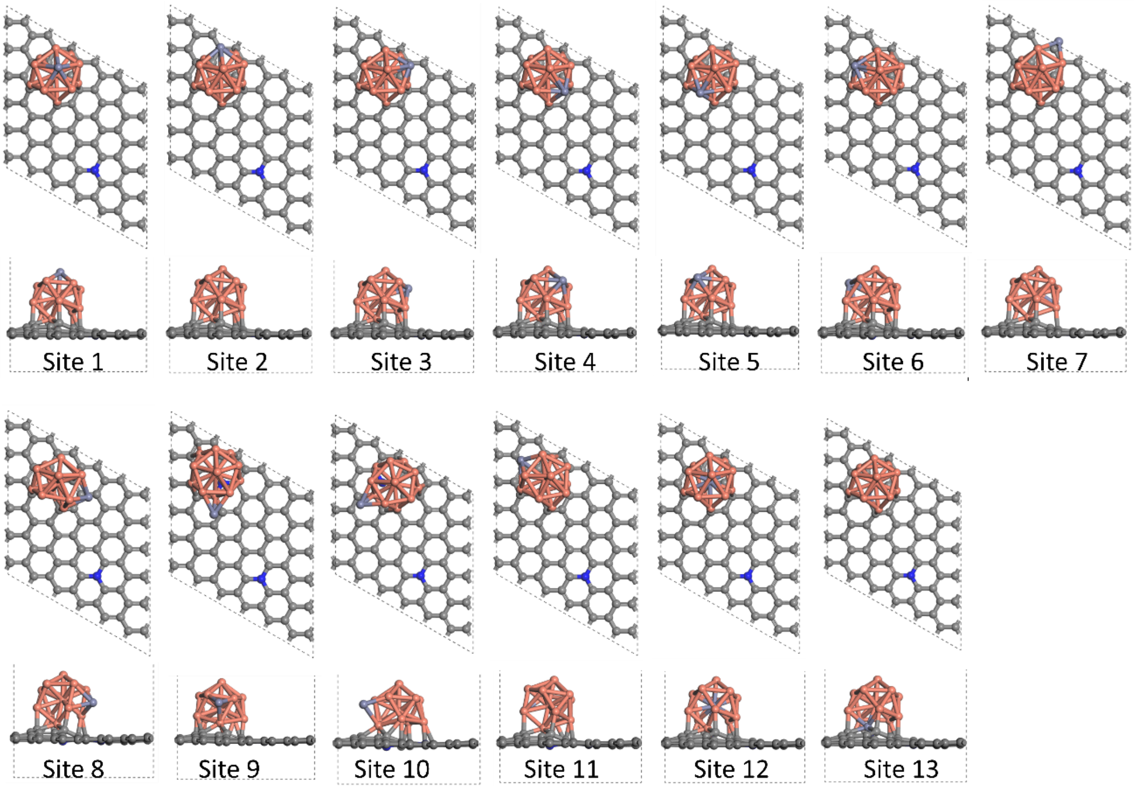

Figure S10. Optimized $Cu_{12}Zn_1$ adsorbed on pyridinic-N of N-doped graphene. Effect of different adsorption sites of $Cu_{12}Zn_1$. Brown, black, silver, and blue represent Cu, C, Zn and N atoms, respectively.

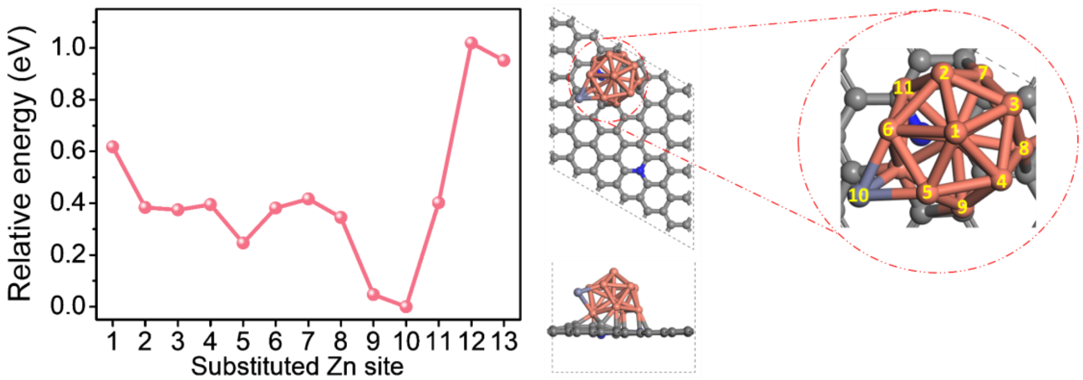

Figure S11. Left: energy of optimized $Cu_{12}Zn_1$ cluster adsorbed on pyridinic-N of N-doped graphene. Effect of different adsorption sites of $Cu_{12}Zn_1$ (see Figure S9). Right: detail (from Figure 92) of the most stable (Zn at site-10) $Cu_{12}Zn_1$ cluster adsorbed on pyridinic-N of N-doped graphene, which will be considered for the reaction paths.

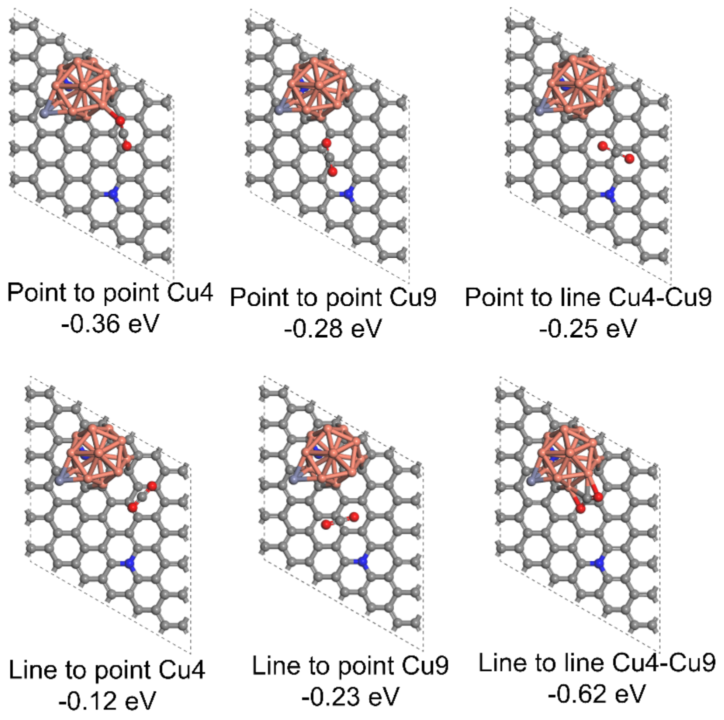

Figure S12. Optimized structure of various CO_2 adsorption configurations on $Cu_{12}Zn_1$ adsorbed on pyridinic-N of N-doped graphene. Brown, black, silver, red and blue represent Cu, C, Zn, O and N atoms, respectively. 'Point to point' refers to the vertical adsorption of CO_2 in Cu sites. 'Point to line' refers to the vertical adsorption of CO_2 into Cu-Cu bridge sites. 'Line to point' refers to the parallel adsorption of CO_2 into Cu sites. 'Line to line' refers to the parallel adsorption of CO_2 in Cu-Cu sites.

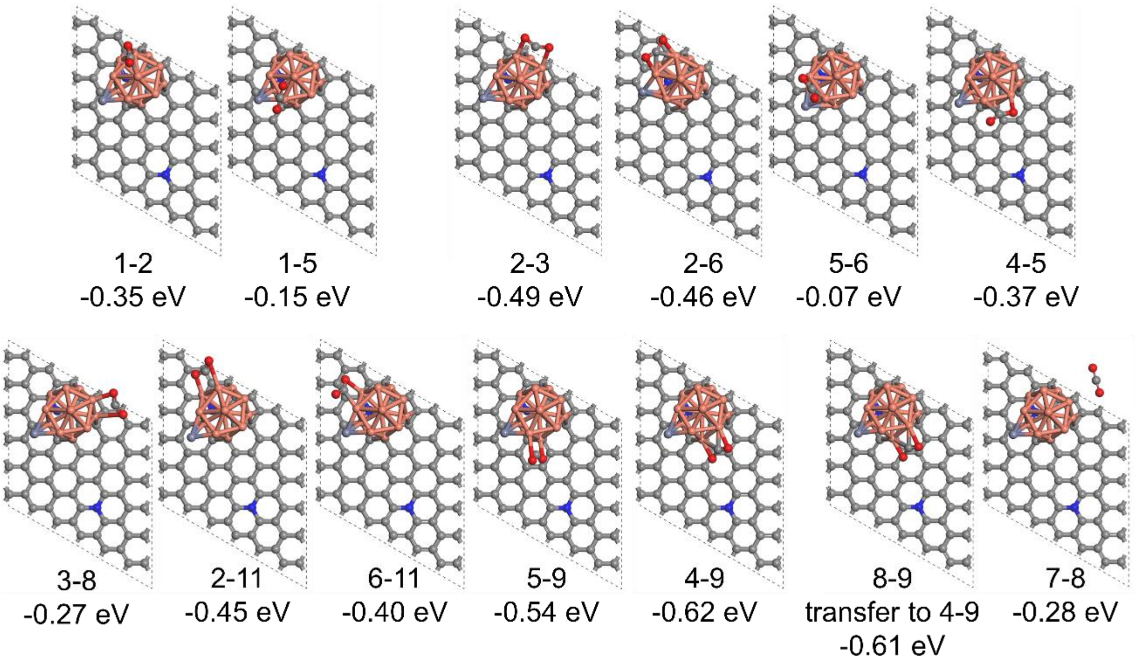

Figure S13. Optimized structure of various CO_2 adsorption sites on $Cu_{12}Zn_1$ adsorbed on pyridinic-N of N-doped graphene. CO_2 interacts with Cu-Cu sites in $Cu_{12}Zn_1$, whose atom numbers are indicated. Corresponding adsorption energies in eV indicated in each case. Brown, black, silver, red and blue represent Cu, C, Zn, O and N atoms, respectively.

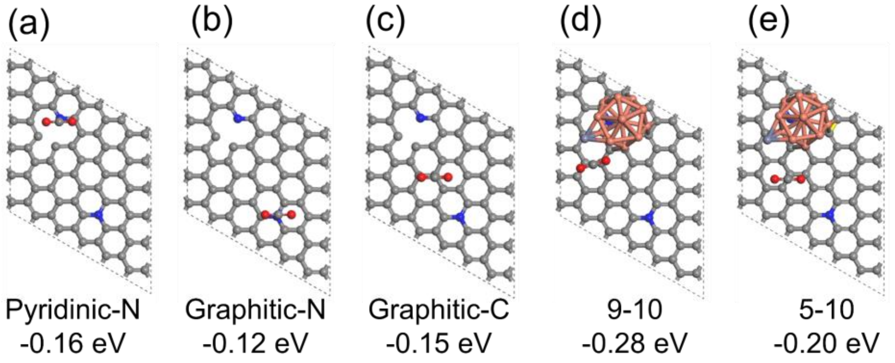

Figure S14. Optimized structure of various CO_2 adsorption sites on different sites (a-c) of N-doped graphene, without and with $Cu_{12}Zn_1$ (d-e) adsorbed on pyridinic-N. CO_2 interacts with N-C, C-C or Cu-Zn sites. The brown, black, silver, red and blue represent Cu, C, Zn, O and N atoms, respectively.

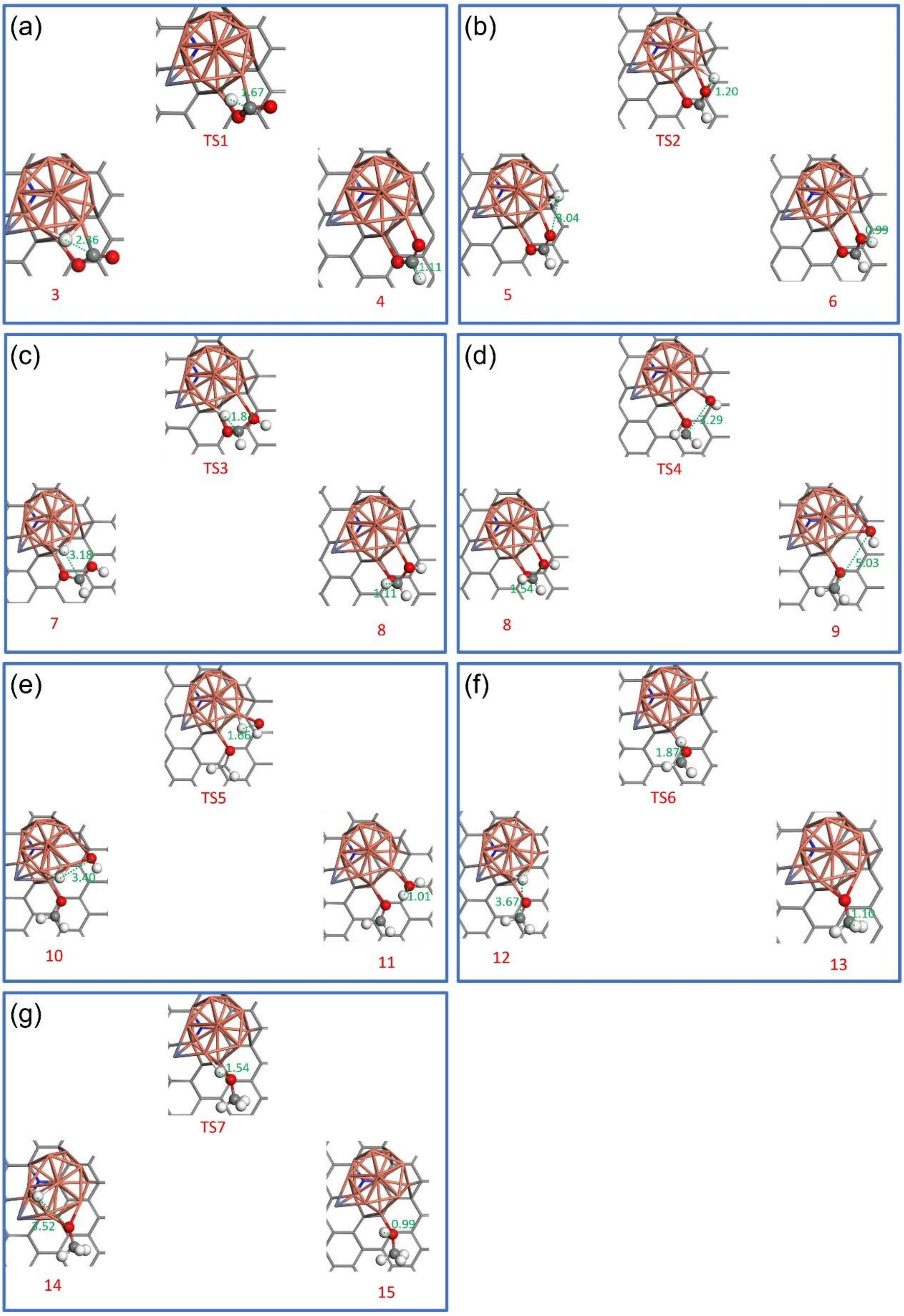

Figure S15. Optimized structures of reactants, transition states and products of all steps of the hydrogenation of CO_2 to methanol following the formate pathway. Energies and elementary steps indicated in Figure 5 and Table S14 respectively.

Notation of intermediates taken from Figure 2. All distances in Å.

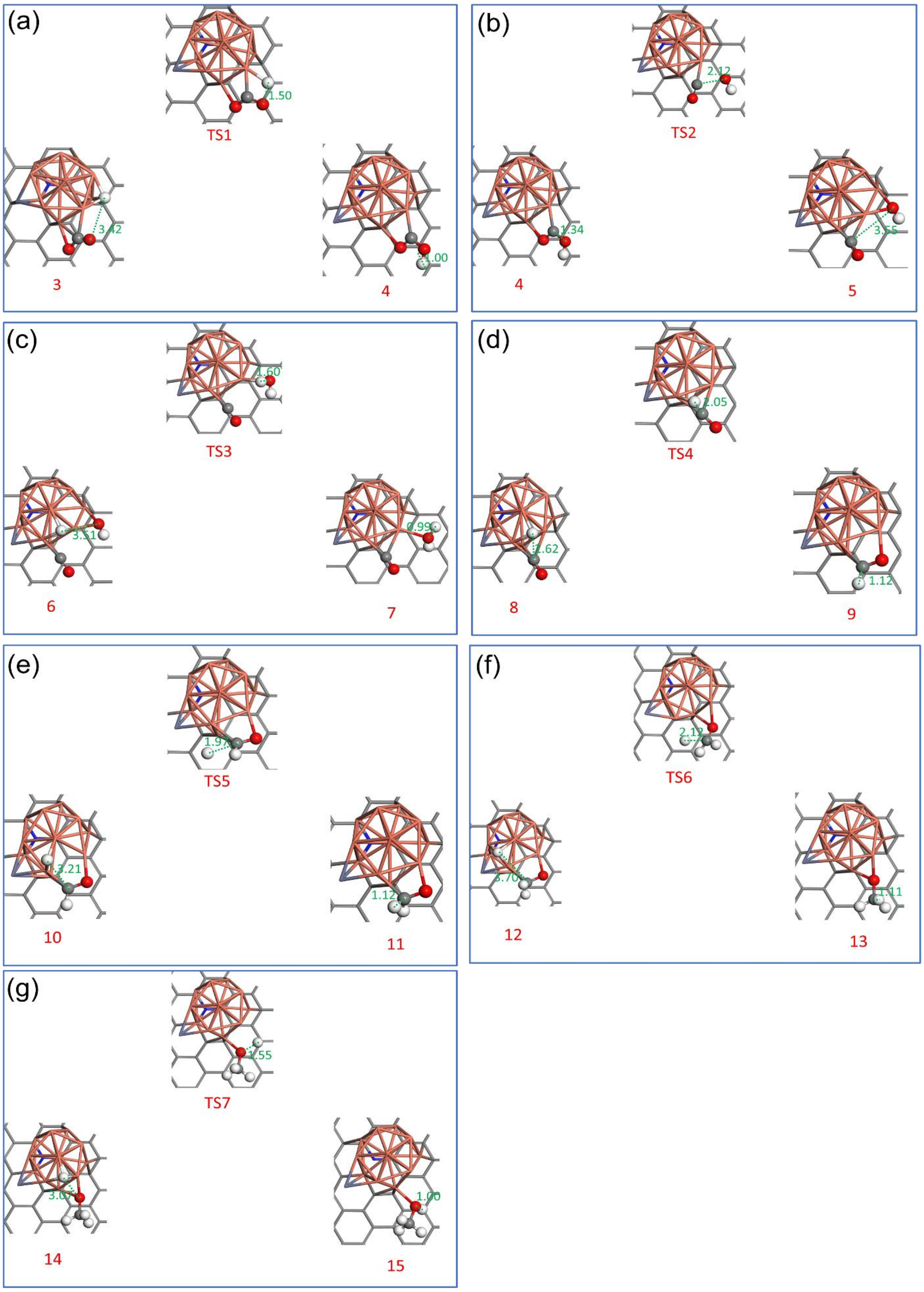

Figure S16. Optimized structures of reactants, transition states and products of all steps of the hydrogenation of CO_2 to methanol following the RWGS+CO-hydro pathway. Energies and elementary steps indicated in Figure 5 and Table S14 respectively.

Notation of intermediates taken from Figure 2. All distances in Å.

References

[1] M. Segall, P.J. Lindan, M.a. Probert, C.J. Pickard, P.J. Hasnip, S. Clark, M. Payne, First-principles simulation: ideas, illustrations and the CASTEP code, Journal of physics: condensed matter, 14 (2002) 2717.

[2] J.P. Perdew, A. Ruzsinszky, G.I. Csonka, O.A. Vydrov, G.E. Scuseria, L.A. Constantin, X. Zhou, K. Burke, Restoring the density-gradient expansion for exchange in solids and surfaces, Physical review letters, 100 (2008) 136406.

[3] A. Tkatchenko, M. Scheffler, Accurate molecular van der Waals interactions from ground-state electron density and free-atom reference data, Physical review letters, 102 (2009) 073005.

[4] T.A. Halgren, W.N. Lipscomb, The synchronous-transit method for determining reaction pathways and locating molecular transition states, Chemical Physics Letters, 49 (1977) 225-232.

[5] A. Primo, J. He, B. Jurca, B. Cojocaru, C. Bucur, V.I. Parvulescu, H. Garcia, CO2 methanation catalyzed by oriented MoS2 nanoplatelets supported on few layers graphene, Applied Catalysis B: Environmental, 245 (2019) 351-359.

[6] H. Fogler, Diffusion and reaction, Elements of chemical reaction engineering, (2006) 813-852.

[7] M.A. Vannice, W.H. Joyce, Kinetics of catalytic reactions, Springer, 2005.

[8] J.O. Hirschfelder, C.F. Curtiss, R.B. Bird, Molecular theory of gases and liquids, Molecular theory of gases and liquids, (1964).

[9] J. Zhu, Y. Su, J. Chai, V. Muravev, N. Kosinov, E.J. Hensen, Mechanism and nature of active sites for methanol synthesis from CO/CO2 on Cu/CeO2, ACS Catalysis, 10 (2020) 11532-11544.

[10] C. Temvuttirojn, Y. Poo-arporn, N. Chanlek, C.K. Cheng, C.C. Chong, J. Limtrakul, T. Witoon, Role of calcination temperatures of ZrO2 support on methanol synthesis from CO2 hydrogenation at high reaction temperatures over ZnO x/ZrO2 catalysts, Industrial & Engineering Chemistry Research, 59 (2020) 5525-5535.

[11] K. Oshima, Y. Honma, K. Kinoshita, Z. Gao, T. Honma, S. Tada, S. Satokawa, Mechanochemical effect in mixing sponge copper with amorphous ZrO2 creates effective active sites for methanol synthesis by CO2 hydrogenation, The Journal of Physical Chemistry C, 125 (2021) 8155-8162.

[12] S. Ghosh, J. Sebastian, L. Olsson, D. Creaser, Experimental and kinetic modeling studies of methanol synthesis from CO2 hydrogenation using In2O3 catalyst, Chemical Engineering Journal, 416 (2021) 129120.

[13] S. Chen, J. Zhang, F. Song, Q. Zhang, G. Yang, M. Zhang, X. Wang, H. Xie, Y. Tan, Induced high selectivity methanol formation during CO2 hydrogenation over a CuBr2-modified CuZnZr catalyst, Journal of catalysis, 389 (2020) 47-59.

[14] M. Mureddu, S. Lai, L. Atzori, E. Rombi, F. Ferrara, A. Pettinau, M.G. Cutrufello, Ex-LDH-based catalysts for CO2 conversion to methanol and dimethyl ether, Catalysts, 11 (2021) 615.
